# Simultaneous Splenectomy and Partial Hepatectomy for Hepatocellular Carcinoma in a Cirrhotic Patient With Severe Hypersplenism: A Case Report

**DOI:** 10.7759/cureus.100951

**Published:** 2026-01-06

**Authors:** Tomoaki Daio, Yujo Kawashita, Shintaro Hirayama, Masaki Tateishi, Takashi Ueda, Junzo Yamaguchi, Masashi Haraguchi, Kouya Umeda, Masayuki Nakamura, Seiko Harada, Sosei Abe, Yoichi Hachitanda

**Affiliations:** 1 Surgery, Fukuoka Seishukai Hospital, Fukuoka, JPN; 2 Pathology, Fukuoka Seishukai Hospital, Fukuoka, JPN

**Keywords:** hepatocellular carcinoma, hypersplenism, live cirrhosis, partial hepatectomy, portal hypertension, splenectomy, thrombocytopenia

## Abstract

Hepatocellular carcinoma (HCC) often arises in the setting of liver cirrhosis and portal hypertension, which complicates curative treatment strategies. Although liver transplantation represents the gold standard treatment for HCC in cirrhotic patients, severe organ shortage, particularly in Asian countries including Japan, limits its availability. Hypersplenism associated with massive splenomegaly frequently results in severe thrombocytopenia, limiting the feasibility of surgical resection. We report the case of a 55-year-old man with alcohol-related cirrhosis, portal hypertension, and marked pancytopenia who was diagnosed with primary HCC. Liver transplantation was discussed, but was not feasible due to the absence of potential living donors and the prolonged waiting time for deceased donor transplantation in Japan. Imaging revealed a 2-cm hypervascular tumor in segment five of the liver and massive splenomegaly with multiple collateral vessels. Given profound thrombocytopenia (platelet count 32,000/µL), simultaneous splenectomy and partial hepatectomy were performed. The surgery proceeded safely despite advanced cirrhosis (Child-Pugh B, Indocyanine green retention test (ICG-R15) 36%), with perioperative transfusion support. Postoperatively, platelet counts improved rapidly, peaking at 310,000/µL, and liver function stabilized without major complications. Histopathology confirmed moderately differentiated HCC (pT2N0M0, stage II). At the three-month follow-up, no recurrence or portal vein thrombosis was detected, and the patient resumed normal activities. This case highlights the potential role of concomitant splenectomy in selected cirrhotic patients with hypersplenism who are not candidates for liver transplantation, enabling safe hepatic resection and possibly promoting hepatic regeneration through hematologic and hemodynamic mechanisms. However, careful patient selection and vigilant management of splenectomy-related risks remain essential.

## Introduction

Hepatocellular carcinoma (HCC) represents the sixth most common cancer worldwide and the third leading cause of cancer-related mortality [1]. The majority of HCC cases develop in the context of chronic liver disease and cirrhosis [2]. The underlying etiologies include chronic hepatitis B and C virus infection, alcohol-related liver disease, non-alcoholic steatohepatitis, and other causes of chronic hepatic inflammation. The progression from chronic hepatitis to cirrhosis creates a microenvironment conducive to hepatocarcinogenesis through repeated cycles of hepatocyte injury, inflammation, and regeneration [2].

Liver transplantation is considered the gold standard treatment for HCC in cirrhotic patients, as it addresses both the malignancy and the underlying liver disease, providing superior long-term oncologic outcomes compared to resection [3]. However, liver transplantation is limited by the availability of donor organs. In Western countries, deceased donor liver transplantation (DDLT) is the primary modality. In contrast, in Japan and other Asian countries, severe organ shortage has resulted in living donor liver transplantation (LDLT) accounting for more than 95% of all liver transplants [4]. The rate of deceased organ donations in Japan remains at less than 1 per million population, compared to approximately 20-40 per million in Western countries. This critical shortage means that many patients with HCC and cirrhosis cannot access transplantation and require alternative therapeutic strategies.

Surgical resection remains an important curative treatment option for patients who are not candidates for liver transplantation; however, portal hypertension and hypersplenism in cirrhotic patients often result in severe thrombocytopenia, posing a major barrier to safe surgical intervention [5,6].

Splenectomy has been proposed to ameliorate portal hypertension, correct cytopenia, and potentially promote hepatic regeneration through hemodynamic improvement and increased platelet-derived growth factors [7-9]. Despite these benefits, splenectomy carries inherent risks including infection and portal vein thrombosis, necessitating careful patient selection [10].

Recent studies suggest that simultaneous splenectomy and hepatectomy may be a feasible strategy in carefully selected cirrhotic patients with HCC and hypersplenism [11,12]. We present a case of alcohol-related cirrhosis with severe thrombocytopenia and massive splenomegaly, in which simultaneous splenectomy and partial hepatectomy allowed for safe and curative resection of HCC in a patient who was not a candidate for liver transplantation. We also provide a review of the literature regarding the mechanisms, benefits, and risks of concomitant splenectomy in cirrhotic patients undergoing hepatic resection for HCC.

## Case presentation

Patient information and clinical findings

A 55-year-old man with a history of alcohol-related cirrhosis was referred to our hospital after esophagogastroduodenoscopy during routine screening revealed esophageal and gastric varices. He had abstained from alcohol for four years but had a prior history of consuming approximately 1 liter of beer daily for more than 20 years. Physical examination revealed palpable hepatomegaly (two finger-breadths below the costal margin) and bilateral gynecomastia, without jaundice or ascites. Notably, neither clinical nor imaging evidence of ascites was present. His medical history was otherwise unremarkable, with no known drug allergies or previous surgical interventions. The patient was unmarried and had no living relatives (parents deceased, no siblings or children).

Diagnostic assessment

Laboratory studies showed elevated tumor markers with alpha-fetoprotein (AFP) at 12.2 ng/mL (normal: 0-10.0 ng/mL) and protein induced by vitamin K absence-II (PIVKA-II) at 79 mAU/mL (normal: 0-40.0 mAU/mL). Complete blood count revealed severe pancytopenia, most notably thrombocytopenia with a platelet count of 32,000/µL (normal: 120,000-350,000/µL), white blood cell count of 2700/µL (normal: 3300-9000/µL), and hemoglobin of 13.2 g/dL (normal: 13.5-17.5 g/dL). Liver function tests demonstrated mildly elevated total bilirubin at 2.1 mg/dL (normal: 0.30-1.20 mg/dL)(direct bilirubin 0.8 mg/dL, indirect bilirubin 1.3 mg/dL), albumin of 3.5 g/dL (normal: 4.0-5.0 g/dL), and prolonged prothrombin time with PT-INR of 1.22 (normal: 0.85-1.15). Activated partial thromboplastin time (APTT) was 36.2 seconds (normal: 25-40 seconds), and fibrinogen was 186 mg/dL (normal: 150-400 mg/dL). Serum sodium was normal at 138 mEq/L. Indocyanine green retention at 15 minutes (ICG-R15) was elevated at 36% (normal: 0-10%), indicating compromised hepatic functional reserve. Based on these findings, the patient was classified as Child-Pugh class B (score 8) with hepatic functional reserve of grade B according to the Liver Damage Classification (Table 1).

**Table 1:** Laboratory test results Laboratory parameters showing severe thrombocytopenia, pancytopenia, and compromised hepatic functional reserve (Child-Pugh B, ICG-R15 36%). Direct bilirubin was 0.8 mg/dL and indirect bilirubin was 1.3 mg/dL. APTT was 36.2 seconds (normal: 25-40 seconds), and fibrinogen was 186 mg/dL (normal: 150-400 mg/dL). TP - total protein; Alb - albumin; T-Bil - total bilirubin; D-Bil - direct bilirubin; I-Bil - indirect bilirubin; AST - aspartate aminotransferase; ALT - alanine aminotransferase; γ-GTP - gamma-glutamyl transferase; ALP - alkaline phosphatase; BUN - blood urea nitrogen; Cre - creatinine; WBC - white blood cells; RBC - red blood cells; Hb - hemoglobin; Ht - hematocrit; Plt - platelets; AFP - alpha-fetoprotein; PIVKA-II - protein induced by vitamin K absence-II; PT - prothrombin time; APTT - activated partial thromboplastin time; ICG - indocyanine green retention test

Laboratory parameters	values	Normal range
TP	6.1 g/dL	6.7-8.3 g/dL
Alb	3.5 g/dL	4.0-5.0 g/dL
T-Bil	2.1 mg/dL	0.30-1.20 mg/dL
AST	26 U/L	13-33 U/L
ALT	17 U/L	6-30 U/L
γ-GTP	17 U/L	10-47 U/L
ALP	101 U/L	38-113 U/L
T-cho	145 mg/dL	120-219 mg/dL
TG	39 mg/dL	30-149 mg/dL
BUN	15.4 mg/dL	8.0-22.0 mg/dL
Cre	0.59 mg/dL	0.60-1.10 mg/dL
Sodium	138 mEq/L	138-146 mEq/L
Potassium	4.2 mEq/L	3.6-4.9 mEq/L
Chloride	107 mEq/L	99-109 mEq/L
WBC	2700 /μL	3300-9000 /μL
%Neut	67.1%	
%Lymph	20.0%	
%Mono	8.9%	
%Eosio	3.9%	
%Baso	0.3%	
RBC	411 ×10⁴/μL	430-570 ×10⁴/μL
Hb	13.2 g/dL	13.5-17.5 g/dL
Ht	37.6%	42.0-53.0%
Plt	3.2 ×10⁴/μL	1.20-3.50 ×105/μL
AFP	12.2 ng/mL	0-10.0 ng/mL
PIVKA-Ⅱ	79 mAU/mL	0-40.0 mAU/mL
PT	68.0%	70.0-100.0%
PT-INR	1.22	0.85-1.15
ICG	36%	0-10%

**Figure 1:**
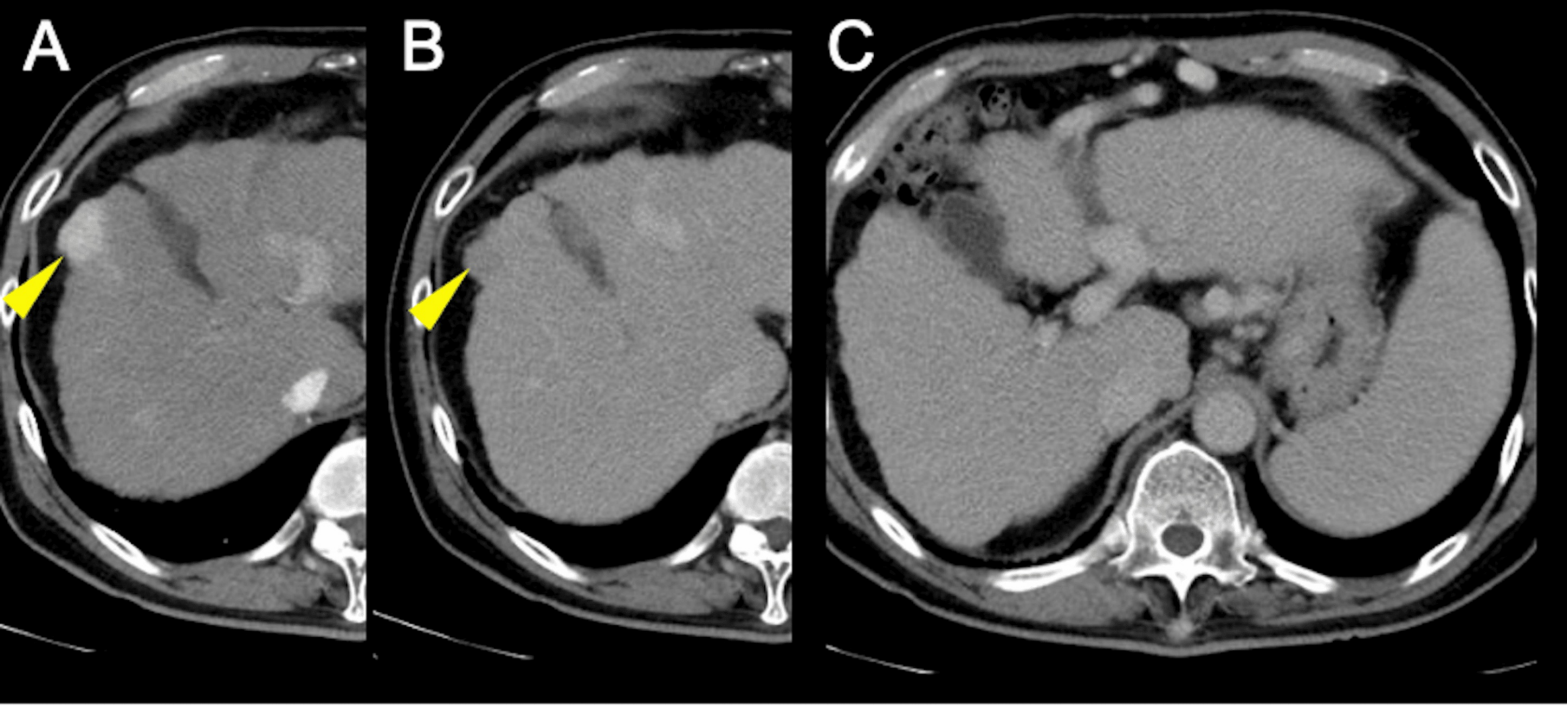
Radiological findings of hepatocellular carcinoma with splenomegaly (A, B) On contrast-enhanced axial CT, a 2-cm lesion in hepatic segment five shows early arterial enhancement and subsequent washout (yellow arrow), consistent with HCC. (C) The spleen is massively enlarged, and multiple collateral vessels are observed around it, indicating advanced portal hypertension. The liver measured approximately 18 cm in craniocaudal dimension at the mid-clavicular line. No free intraperitoneal fluid was detected.

Contrast-enhanced computed tomography (CT) demonstrated a 2-cm hypervascular lesion in segment five of the liver, showing early arterial enhancement with subsequent washout in the portal venous phase, consistent with HCC (Figure 1A, B). CT imaging also demonstrated hepatomegaly with a craniocaudal dimension of approximately 18 cm at the mid-clavicular line, without evidence of free intraperitoneal fluid.

**Figure 2:**
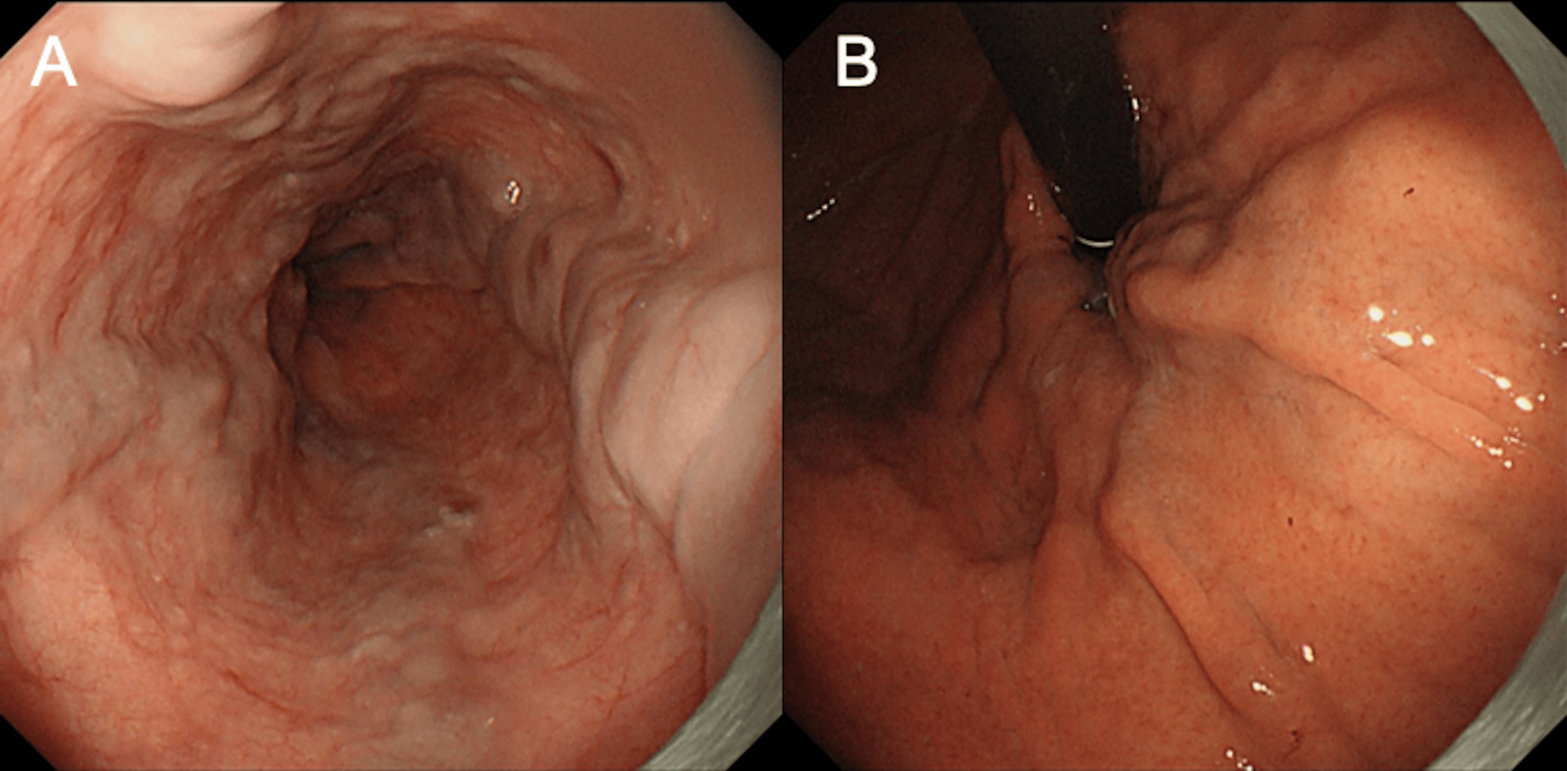
Findings of upper gastrointestinal endoscopy showing the presence of varices (A) Esophageal varices, Lm. F2-3 Cb RC0. (B) Gastric varices, Lg-C. F1-2 Cw RC0.

The imaging also revealed massive splenomegaly with multiple collateral vessels surrounding the spleen, indicating advanced portal hypertension (Figure 1C). Upper gastrointestinal endoscopy confirmed the presence of esophageal varices (F2-3) located in the middle to lower esophagus without red color signs (RC0), as well as gastric varices (F1-2) in the cardiac region without red color signs (Figure 2).

No evidence of extrahepatic metastasis or portal vein tumor thrombosis was identified.

Therapeutic intervention

Treatment options were discussed comprehensively with the patient. Liver transplantation was explained as the gold standard treatment for HCC in cirrhotic patients. However, due to the severe shortage of deceased donor organs in Japan and the absence of potential living donors (the patient was unmarried with no living relatives), liver transplantation was not feasible. The expected prolonged waiting time for deceased donor transplantation in Japan carries a substantial risk of tumor progression and hepatic decompensation. Therefore, simultaneous splenectomy and partial hepatectomy were planned as the most feasible curative option.

Given the patient's profound thrombocytopenia and the need for curative resection of HCC, simultaneous splenectomy and partial hepatectomy were planned preoperatively. While platelet transfusion could provide temporary correction of thrombocytopenia, this approach would not address the underlying hypersplenism, and transfused platelets would be rapidly consumed by the enlarged spleen. Splenectomy offers the advantage of sustained hematologic improvement, reduction in portal pressure, and potential hepatotrophic benefits that cannot be achieved with transfusion alone. Preoperatively, the patient received vaccinations against Streptococcus pneumoniae, Haemophilus influenzae type b, and Neisseria meningitidis to reduce the risk of overwhelming post-splenectomy infection (OPSI).

The procedure was initiated laparoscopically to assess the operative field; however, early conversion to open surgery was anticipated and executed due to the massive spleen (weight: 480 grams) and highly developed collateral circulation, which precluded safe laparoscopic dissection.

**Figure 3:**
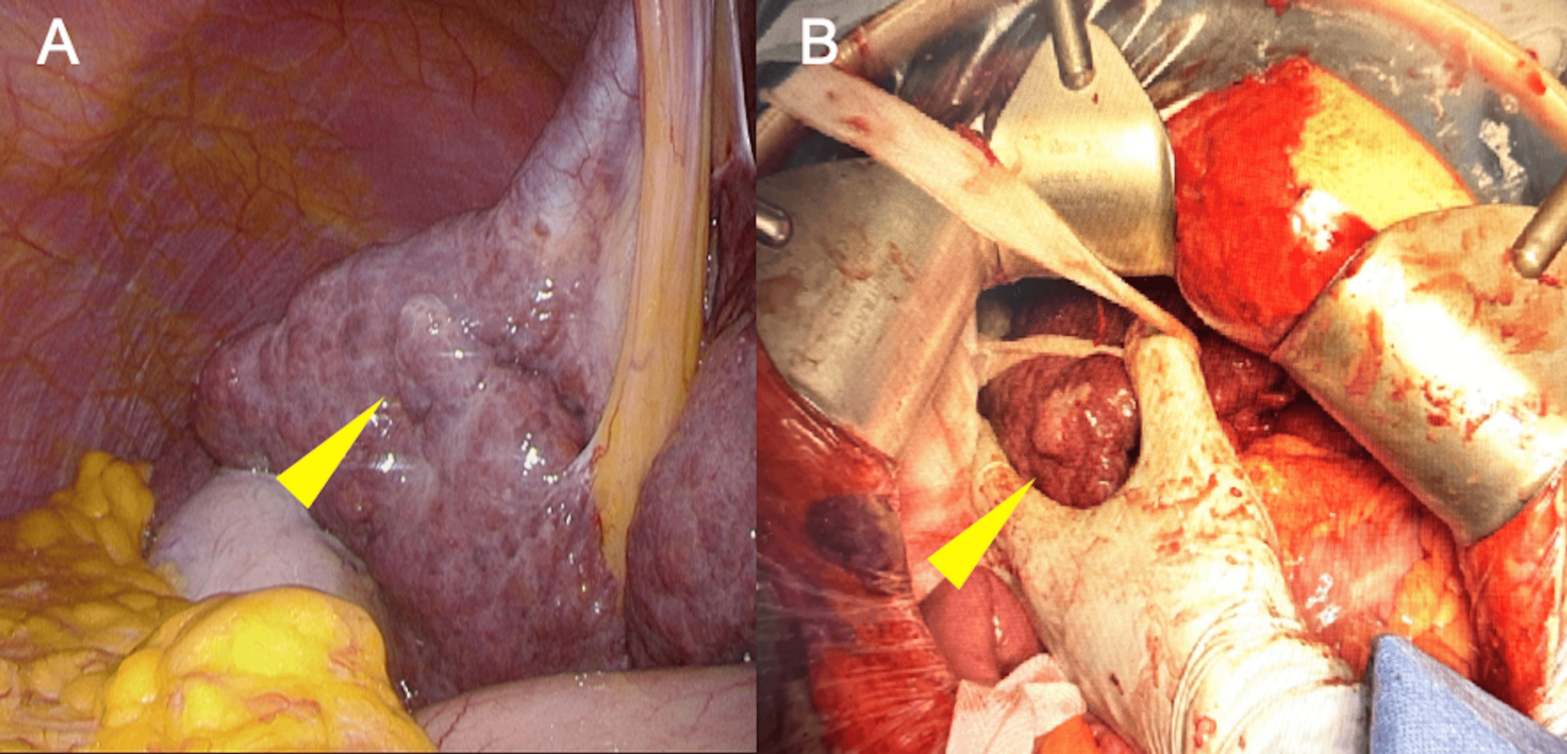
Intraoperative findings (A) Laparoscopic view showing the tumor (yellow arrow) in the cirrhotic liver with nodular surface. (B) Open surgical view after conversion, demonstrating the macroscopic appearance of the tumor (yellow arrow) within the cirrhotic liver parenchyma. Hepatic parenchymal transection was performed using a two-surgeon technique with the Sonopet ultrasonic aspirator and ball-type electrode for hemostasis.

Hemostasis for the subsequent hepatic resection. Multiple collateral vessels around the splenic hilum were carefully ligated. Following splenectomy, partial resection of liver segment five was performed using a two-surgeon technique with the Sonopet ultrasonic aspirator (Stryker, Kalamazoo, Michigan) for parenchymal transection and ball-type electrode for hemostasis, combined with intermittent Pringle maneuver. Intraoperative findings revealed a cirrhotic liver with nodular surface and a firm, well-demarcated tumor measuring approximately 2 cm in diameter (Figure 3). Closed suction drains were placed at the hepatic resection site and splenic fossa.

Perioperative transfusion included 10 units of platelet concentrate, 12 units of red blood cells, and 480 mL of fresh frozen plasma. The operation was completed in approximately 2.5 hours with an estimated blood loss of 850 mL.

Follow-up and outcomes

**Figure 4:**
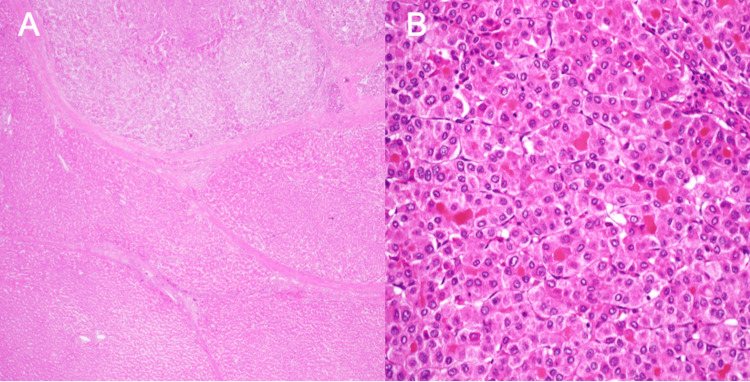
Pathological findings Postoperative pathological examination revealed moderately differentiated hepatocellular carcinoma within regenerative nodules. Both preserved and disrupted glandular structures were observed within the lesion. (A) Low-power view showing tumor within cirrhotic parenchyma. (B) High-power view showing trabecular growth pattern.

Histopathological examination of the resected specimen revealed moderately differentiated hepatocellular carcinoma arising within a cirrhotic liver parenchyma. The tumor showed a trabecular growth pattern with preserved and disrupted glandular structures (Figure 4).

Surgical margins were negative, and no vascular invasion was identified. The final pathological staging was pT2N0M0 (stage II) according to the TNM classification.

**Figure 5:**
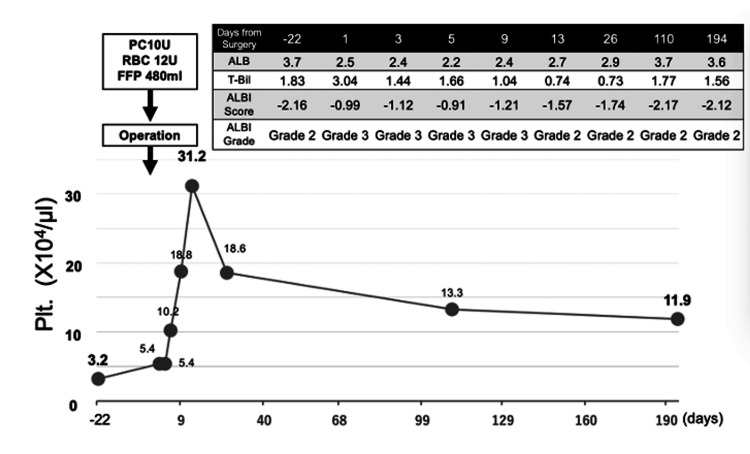
Clinical course and changes in platelet count with liver injury markers After peaking in the early postoperative period (310,000/µL on day nine), the platelet count remained stable within the normal range (110,000-130,000/µL at six months follow-up). The table shows liver function parameters, including ALB, T-Bil, and ALBI score at various time points. The transient rise in bilirubin to 3.04 mg/dL on postoperative day one did not meet criteria for PHLF according to the ISGLS definition. ALBI score transiently worsened to grade 3 in the immediate postoperative period but improved to grade 2 by postoperative day 13 and remained stable thereafter. The graph shows preoperative preparation with platelet and blood transfusion (PC 10U, RBC 12U, FFP 480ml), followed by dramatic postoperative platelet recovery. ALB - albumin; t-Bil - total bilirubin; ALBI - albumin-bilirubin; PHLF - post-hepatectomy liver failure; ISGLS - International Study Group of Liver Surgery; PC - platelet count; RBC - red blood cell count; FFP - fresh frozen plasma

Postoperatively, platelet counts rose promptly from the preoperative level of 32,000/µL to 54,000/µL on postoperative day one, peaking at 310,000/µL by postoperative day nine. The platelet count subsequently stabilized at approximately 110,000-130,000/µL at six months follow-up, remaining within the normal range (Figure 5). Serum albumin normalized to 3.7 g/dL within one month, and total bilirubin, which transiently increased to 3.04 mg/dL on postoperative day one, rapidly declined to baseline levels. The ALBI (albumin-bilirubin) score, which had transiently worsened to grade 3 in the immediate postoperative period, improved to grade 2 by postoperative day 13 and remained stable thereafter (Figure 5).

**Figure 6:**
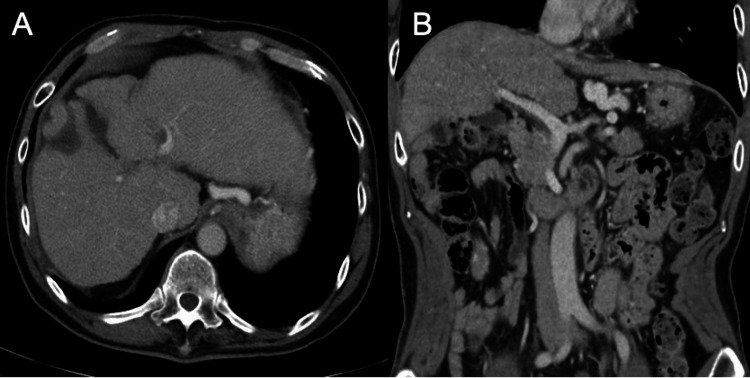
Findings of follow-up CT at three months postoperatively No recurrence of hepatocellular carcinoma was observed. The liver parenchyma shows stable appearance without evidence of new lesions or vascular complications.

The patient experienced no major complications during the postoperative course, including no evidence of portal vein thrombosis, postoperative infection, or liver failure. Anticoagulation therapy with low-molecular-weight heparin was administered prophylactically for two weeks to prevent thromboembolic complications. The patient was discharged on postoperative day 14 in good condition. At three-month follow-up, CT scans showed no recurrence of HCC, no vascular complications, and the patient remained asymptomatic (Figure 6).

He had resumed his normal daily activities and returned to work. Continued surveillance imaging and tumor marker monitoring are planned according to standard follow-up protocols for HCC.

## Discussion

Hepatocellular carcinoma developing in cirrhotic patients presents unique therapeutic challenges, particularly when complicated by portal hypertension and hypersplenism. Liver transplantation represents the gold standard treatment for HCC in cirrhotic patients, as it addresses both the malignancy and the underlying liver disease, providing superior long-term oncologic outcomes compared to resection [3]. Meta-analyses have demonstrated that liver transplantation is associated with significantly better five-year overall survival (64.83%) and recurrence-free survival (70.20%) compared to liver resection (overall survival: 50.83%; recurrence-free survival: 34.46%). However, the availability of liver transplantation is severely limited by organ shortage, particularly in Asian countries. In Japan, the rate of deceased organ donations remains at less than one per million population, and living donor liver transplantation (LDLT) accounts for more than 95% of all liver transplants [4]. LDLT requires a healthy relative or close acquaintance willing to undergo partial hepatectomy, making this option unavailable to patients without suitable donors.

In our patient, liver transplantation was discussed as part of comprehensive treatment planning. However, the patient was unmarried with no living relatives who could serve as potential living donors, and the expected prolonged waiting time for deceased donor transplantation carried a substantial risk of tumor progression and hepatic decompensation. Given these constraints, simultaneous splenectomy and hepatectomy were selected as the most feasible curative option.

For patients who cannot undergo liver transplantation, surgical resection remains an important curative option. However, the presence of severe thrombocytopenia not only increases perioperative bleeding risk but also serves as a marker of advanced portal hypertension, limiting the applicability of curative treatments [5]. Traditional management strategies have focused on treating HCC and portal hypertension separately; however, emerging evidence suggests that addressing both conditions simultaneously may yield superior outcomes in selected patients [11,12]. The reported prevalence of thrombocytopenia (platelet count <100,000/µL) in cirrhotic patients ranges from 64% to 84%, with severe thrombocytopenia (<50,000/µL) observed in 13-15% of cases [13]. This cytopenia arises from multiple mechanisms, including splenic sequestration, decreased thrombopoietin production by the diseased liver, bone marrow suppression, and increased platelet consumption [7]. In our patient, profound thrombocytopenia (32,000/µL) posed a significant barrier to safe surgical intervention, necessitating a combined approach to address both the malignancy and the underlying hypersplenism.

Splenectomy in cirrhotic patients exerts multiple beneficial effects through interconnected hemodynamic, hematologic, and hepatotrophic mechanisms, supported by extensive experimental and clinical evidence. The procedure reduces portal venous inflow by eliminating splenic blood flow, which typically accounts for 20-40% of total portal blood flow [14]. This reduction in portal venous inflow leads to decreased portal pressure and improved hepatic hemodynamics. Liang et al. (2021) demonstrated in a murine model that splenectomy significantly ameliorated liver fibrosis through modulation of the tumor necrosis factor superfamily member 14 (LIGHT)/JNK/TGF-β1 signaling pathway [8]. The reduction in intrahepatic shear stress following splenectomy promotes recovery of sinusoidal endothelial cell function, leading to increased nitric oxide production and improved hepatic microcirculation. Furthermore, splenectomy removes a significant source of vasoactive mediators, including endothelin-1 and tumor necrosis factor-alpha, which contribute to increased intrahepatic vascular resistance in cirrhosis. Clinical studies have consistently demonstrated that splenectomy results in a 15-30% reduction in portal pressure, although the effect may be less pronounced in patients with advanced cirrhosis and extensive portosystemic collaterals [15].

The immediate hematologic benefit of splenectomy is correction of cytopenia, particularly thrombocytopenia. In our case, platelet count increased nearly tenfold within two weeks postoperatively, facilitating safe surgical intervention and reducing bleeding risk. However, the role of platelets extends far beyond hemostasis. Platelets are now recognized as critical mediators of liver regeneration through the release of multiple growth factors and cytokines [9,16]. Kurokawa et al. (2017) provided a comprehensive review of the multifaceted roles of platelets in liver disease, demonstrating that platelets contain and release hepatocyte growth factor (HGF), vascular endothelial growth factor (VEGF), insulin-like growth factor-1 (IGF-1), and serotonin [9]. HGF is perhaps the most potent hepatotrophic factor, promoting hepatocyte proliferation, inhibiting apoptosis, and enhancing liver regeneration. Serotonin acts synergistically with HGF to stimulate liver regeneration through the 5-HT2 receptor pathway. Clinical studies have demonstrated that patients with higher platelet counts following liver resection experience faster liver regeneration and better preservation of hepatic function [16].

Additionally, platelets play a crucial role in sinusoidal remodeling and angiogenesis during liver regeneration. Through the release of VEGF and platelet-derived growth factor (PDGF), platelets promote sinusoidal endothelial cell proliferation and restoration of the hepatic microvasculature [9]. This angiogenic response is essential for supporting regenerating hepatocytes and preventing ischemic injury in the remnant liver.

Beyond hemodynamic and hepatotrophic mechanisms, splenectomy may exert direct anti-fibrotic effects on the cirrhotic liver. Yoshida et al. (2014) demonstrated that platelet-derived PDGF-β promotes activation of hepatic stellate cells (HSCs), the primary effector cells of liver fibrosis [17]. Platelets can influence HSC activation and liver fibrosis through multiple signaling pathways, including TGF-β and PDGF [17]. However, the net effect of platelets on fibrosis progression appears to be context-dependent and may vary with platelet count and activation status. Liang et al. (2021) showed that splenectomy improved liver fibrosis in a carbon tetrachloride-induced cirrhosis model through downregulation of LIGHT expression and inhibition of the JNK/TGF-β1 signaling cascade [8]. LIGHT is a pro-inflammatory cytokine highly expressed in the spleen that promotes HSC activation and collagen deposition. By removing this splenic source of LIGHT, splenectomy attenuates ongoing fibrogenesis and may even promote fibrolysis through activation of matrix metalloproteinases. These findings suggest that splenectomy may not only facilitate immediate surgical intervention but also slow or reverse fibrosis progression in the long term, although clinical validation of these effects in humans remains limited.

The concept of performing simultaneous splenectomy and hepatectomy for HCC in cirrhotic patients with hypersplenism has gained increasing attention in recent years. Shi et al. (2021) conducted a systematic review and meta-analysis examining the outcomes of splenectomy in HCC patients with hypersplenism [12]. Their analysis included 1547 patients from 14 studies and demonstrated that splenectomy significantly improved platelet count (weighted mean difference: 107.41 × 10⁹/L, p<0.001) and was associated with improved overall survival (hazard ratio: 0.71, 95% CI: 0.55-0.91, p=0.007) compared to hepatectomy alone. Importantly, the incidence of major complications was not significantly increased in the splenectomy group, suggesting that the procedure can be performed safely in selected patients. The timing of splenectomy relative to hepatectomy remains a subject of debate. Several groups have discussed the merits of simultaneous versus staged procedures, noting that simultaneous surgery offers the advantage of a single anesthetic exposure and may provide immediate hematologic improvement to facilitate hepatic resection. However, staged procedures may be preferable in patients with marginal hepatic reserve or when extensive hepatic resection is required, as they allow for assessment of hepatic functional recovery after splenectomy before proceeding with hepatectomy.

In our case, simultaneous splenectomy and partial hepatectomy proved to be a safe and effective strategy. The patient's rapid hematologic recovery and stable hepatic function in the postoperative period support the feasibility of this approach in carefully selected patients. The decision to perform simultaneous surgery was based on several factors: (1) the patient's relatively preserved hepatic reserve despite Child-Pugh B classification; (2) the limited extent of hepatic resection required for a small, peripherally located tumor; (3) the severity of thrombocytopenia precluding safe hepatectomy without splenectomy; (4) the unavailability of liver transplantation due to absence of potential living donors and severe deceased donor shortage in Japan; and (5) the patient's good performance status and absence of significant comorbidities.

Despite the potential benefits of splenectomy, clinicians must remain cognizant of several serious complications that can occur following splenic removal. The most feared complication is overwhelming post-splenectomy infection (OPSI), a life-threatening condition characterized by fulminant sepsis, most commonly caused by encapsulated organisms such as *Streptococcus pneumoniae*, *Haemophilus influenzae*, and *Neisseria meningitidis* [10]. The reported incidence of OPSI ranges from 0.28% to 0.42% per year, with a lifetime risk of approximately 5%. Notably, recent evidence suggests that the effect of splenectomy on infection risk may be stage-dependent, with increased infection risk in compensated cirrhosis (8.06% vs. 5.18%) but potentially decreased risk in decompensated cases (11.35% vs. 22.22%), possibly due to persistence of accessory splenic tissue and compensatory lymphoid hypertrophy [18]. Nevertheless, all patients should receive appropriate vaccinations preoperatively and should be educated about the lifelong risk of OPSI.

Portal vein thrombosis (PVT) represents another significant complication following splenectomy, with reported incidence ranging from 5% to 55% depending on surveillance intensity, imaging modality, and patient characteristics [19]. The dramatic increase in platelet count following splenectomy, combined with alterations in portal hemodynamics, creates a prothrombotic state. Several risk factors for post-splenectomy PVT have been identified, including preoperative platelet count <40,000/µL, splenomegaly >1000 grams, and peak postoperative platelet count >400,000/µL [19]. To mitigate this risk, many centers have adopted routine postoperative anticoagulation protocols, typically using low-molecular-weight heparin for two to four weeks, followed by transition to oral anticoagulation or antiplatelet therapy in high-risk patients. In our institution, we routinely administer prophylactic anticoagulation for two weeks postoperatively and perform Doppler ultrasonography or CT imaging within the first week to screen for early PVT. Additional complications include postoperative pancreatic fistula (3-8% incidence), subphrenic abscess, and thrombocytosis-related thromboembolic events in non-portal vessels [20]. Long-term consequences of splenectomy may include increased risk of cardiovascular events and pulmonary hypertension, although the clinical significance of these associations in cirrhotic patients remains unclear. Careful patient selection, meticulous surgical technique, and vigilant postoperative monitoring are essential to minimize these risks.

The impact of prior splenectomy on future liver transplantation candidacy should also be considered. While splenectomy does not absolutely preclude subsequent liver transplantation, the adhesions and anatomical changes following splenectomy may increase the technical difficulty of transplant surgery. For patients who may potentially become transplant candidates in the future, the decision to perform splenectomy should be made after careful discussion of these implications [4].

Based on current evidence and clinical experience, careful patient selection is paramount to achieving optimal outcomes with simultaneous splenectomy and hepatectomy. This approach should be considered primarily for patients who are not candidates for liver transplantation. Patients should have relatively preserved liver function, ideally Child-Pugh class A, or Child-Pugh class B with good compensatory reserve (score ≤8) and ICG-R15 <40%. Patients with Child-Pugh class C cirrhosis or those with refractory ascites should generally not be considered candidates for this procedure due to prohibitively high perioperative mortality risk. The primary indication for concomitant splenectomy is severe thrombocytopenia (platelet count <50,000/µL) that precludes safe hepatic resection. Patients with moderate thrombocytopenia (50,000-100,000/µL) may be managed with alternative strategies such as platelet transfusion or thrombopoietin receptor agonists. The extent of hepatic resection should be limited to minor or moderate resections (≤3 segments), as extensive hepatectomy combined with splenectomy carries excessive risk of hepatic decompensation. Tumors should ideally be peripherally located, resectable with adequate margin, and without evidence of major vascular invasion or extrahepatic spread. While portal hypertension is a prerequisite for clinically significant hypersplenism, patients with severe portal hypertension and high-risk varices may benefit from prophylactic endoscopic variceal ligation prior to surgery. The presence of significant ascites or hepatic encephalopathy suggests advanced portal hypertension and should prompt careful reassessment of surgical candidacy. Patients should have a good performance status (ECOG 0-1) and adequate cardiopulmonary reserve to tolerate the physiologic stress of combined surgery. Those with uncorrected coagulopathy, active infection, or significant cardiopulmonary disease should be excluded or optimized prior to surgery.

For patients who do not meet criteria for simultaneous splenectomy and hepatectomy, several alternative strategies exist. Transarterial chemoembolization (TACE) and radiofrequency ablation (RFA) represent effective locoregional treatment options that do not require the same degree of hematologic reserve as surgical resection. Laparoscopic hepatectomy, when feasible, may offer reduced surgical stress and faster recovery compared to open approaches. Partial splenic embolization (PSE) has emerged as a less invasive option to improve cytopenias while preserving partial splenic function and reducing OPSI risk [15]. PSE involves selective embolization of 50-70% of the splenic artery branches, leading to splenic infarction and subsequent improvement in platelet count. Studies have shown that PSE can increase platelet counts by 40,000-60,000/µL, which may be sufficient to enable subsequent therapeutic interventions [15]. However, PSE is associated with significant post-embolization syndrome (fever, abdominal pain) in up to 90% of patients, and the durability of hematologic response is variable, with some patients experiencing recurrent thrombocytopenia within 6-12 months. Pharmacologic interventions, including thrombopoietin receptor agonists such as eltrombopag and avatrombopag, represent another option for managing thrombocytopenia in cirrhotic patients [13]. These agents directly stimulate megakaryopoiesis and can increase platelet counts sufficiently to enable procedures in some patients. However, they do not address the underlying portal hypertension or provide the potential hepatotrophic benefits of splenectomy. For patients with adequate hepatic reserve but requiring extensive hepatic resection, a staged approach with splenectomy performed two to four weeks prior to hepatectomy may be preferable. This strategy allows for complete hematologic recovery and reassessment of hepatic functional reserve before proceeding with definitive oncologic surgery.

Despite accumulating evidence supporting the role of splenectomy in cirrhotic patients with HCC and hypersplenism, several important questions remain unanswered. First, the long-term oncologic outcomes of simultaneous splenectomy and hepatectomy compared to hepatectomy alone or alternative treatment modalities require clarification through prospective, randomized controlled trials. Comparison with liver transplantation outcomes in matched cohorts would also be valuable, recognizing the inherent selection bias in patients who undergo hepatectomy versus transplantation. While existing meta-analyses suggest potential survival benefits, selection bias and heterogeneity in patient populations limit the strength of these conclusions [12]. Second, the optimal management of anticoagulation in the perioperative period needs standardization. Current practices vary widely among institutions, and the balance between preventing PVT and avoiding bleeding complications in patients with cirrhosis and portal hypertension remains delicate. Prospective studies examining different anticoagulation protocols, including timing, dosing, and duration, would provide valuable guidance. Third, the potential anti-fibrotic effects of splenectomy observed in animal models require validation in human studies with longitudinal assessment of fibrosis markers and liver stiffness measurements [8]. If confirmed, these benefits could have important implications for patient selection and prognostication. Finally, comparative effectiveness research examining splenectomy versus PSE, with consideration of cost-effectiveness, quality of life, and long-term outcomes, would help clinicians and patients make informed decisions regarding the optimal approach to managing hypersplenism in cirrhotic patients requiring HCC treatment.

This case report has several limitations. As a single case, our experience cannot be generalized to all cirrhotic patients with HCC and hypersplenism. The relatively short follow-up period (three months) limits our ability to assess long-term oncologic outcomes and late complications. Additionally, the patient's alcohol-related cirrhosis may respond differently to splenectomy compared to cirrhosis of other etiologies, such as viral hepatitis or non-alcoholic steatohepatitis. Larger, prospective studies with extended follow-up are needed to define the role of simultaneous splenectomy and hepatectomy in this patient population.

## Conclusions

We successfully treated a cirrhotic patient with hepatocellular carcinoma and severe thrombocytopenia using simultaneous splenectomy and partial hepatectomy. Although liver transplantation represents the gold standard treatment for HCC in cirrhotic patients, this approach may not be feasible for all patients due to organ shortage and lack of suitable donors, particularly in Asian countries where living donor transplantation predominates. For patients who are not candidates for liver transplantation, simultaneous splenectomy and hepatectomy represents a viable alternative that enables curative resection while promoting rapid hematologic recovery and stable hepatic function without major complications. The multifaceted benefits of splenectomy - including hemodynamic improvement, platelet-mediated hepatotrophic effects, and potential anti-fibrotic properties - provide a strong rationale for this approach in carefully selected patients. Ideal candidates include those with Child-Pugh A-B cirrhosis, severe thrombocytopenia (<50,000/µL), lack of liver transplantation candidacy, and peripherally located tumors amenable to limited resection. Vigilant perioperative management, including appropriate vaccinations, prophylactic anticoagulation, and surveillance for complications such as overwhelming post-splenectomy infection and portal vein thrombosis, remains essential to maximize benefits and minimize risks. Future research should focus on defining optimal patient selection criteria and validating the long-term oncologic outcomes of this combined approach through well-designed prospective trials.
